# Microcystin-LR and its health impacts: Chemistry, transmission routes, mechanisms of toxicity and target organs

**DOI:** 10.1016/j.toxrep.2025.101996

**Published:** 2025-03-11

**Authors:** Roshni Rajpoot, Siddharth Rajput, Raj Kumar Koiri

**Affiliations:** Biochemistry Laboratory, Department of Zoology, School of Biological Sciences, Dr. Harisingh Gour Vishwavidyalaya (A Central University), Sagar, Madhya Pradesh 470003, India

**Keywords:** Microcystins, Toxicity, Protein phosphatase, Mechanism, Route of exposure

## Abstract

Microcystin-LR, a hepatotoxin produced by cyanobacteria, poses significant health risks to humans and other animals through various routes of exposure. This review comprehensively explores the chemistry, transmission pathways, mechanisms of toxicity, and target organs affected by MC-LR to provide a detailed understanding of its health impacts on animals and humans. MC-LR exposure occurs through different transmission routes, including ingesting contaminated water and food, algal dietary supplements, direct body contact with harmful algal blooms, and inhalation of aerosolized toxins. In this review, we explored that the toxic effects of MC-LR are mediated through multiple complex mechanisms. A key mechanism of its toxicity is the inhibition of protein phosphatases PP1 and PP2A which results in abnormal cellular signalling pathways. Additionally, MC-LR induces oxidative stress and disrupts cellular homeostasis. The findings suggest that MC-LR modulates the activity of various antioxidant enzymes and also activates apoptosis pathways by different mechanisms. It also induces cytoskeletal disruption, ultimately compromising cellular integrity and function. MC-LR also induces activation of oncogenes such as Gankyrin, PI3K/AKT, HIF-1α, RAC1/JNK and NEK2 pathway and upregulates the inflammatory molecules such as NF-κβ, and TNF-α, hence leading to carcinogenesis. MC-LR has toxicological effects on multiple organs**.** The liver is the primary target, where MC-LR accumulates and causes hepatotoxicity, but other organs are affected as well. MC-LR shows neurotoxicity, nephrotoxicity, cardiotoxicity and reproductive toxicity.

## Highlights

• In the MC family, Microcystin-leucine arginine (MC-LR) is the most common and toxic compound.
• MC-LR causes toxicity in different organs such as the liver, brain, kidney, lung, intestine, heart and gonads.
• MC-LR toxicity mechanisms involves PP1/PP2A inhibition, oxidative stress, oncogenic pathways, and cytoskeleton disruption.
• Long-term exposure to low concentrations of MCs increases the risk of cancer development.

## Graphical Abstract

**Figure:**
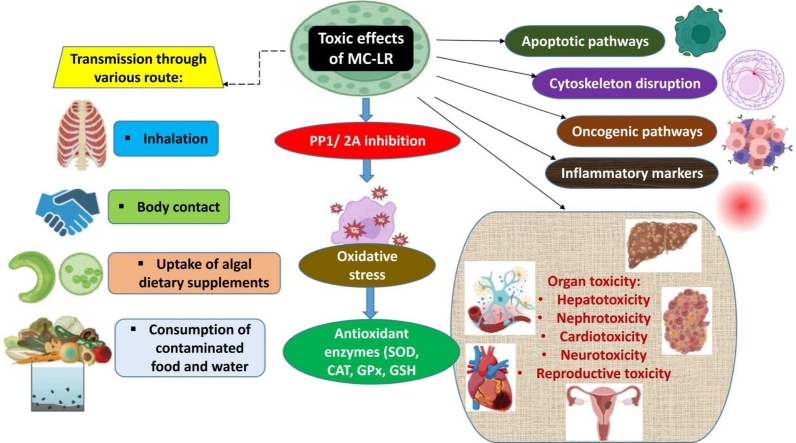

## 1 Introduction

**Fig. 1:**
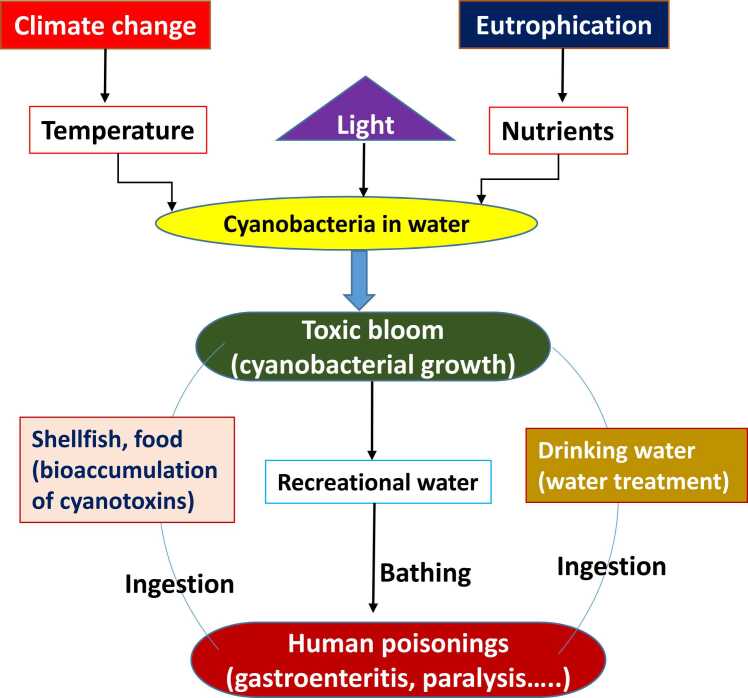
Origin of toxic cyanobacterial blooms and human exposure.

**Fig. 2:**
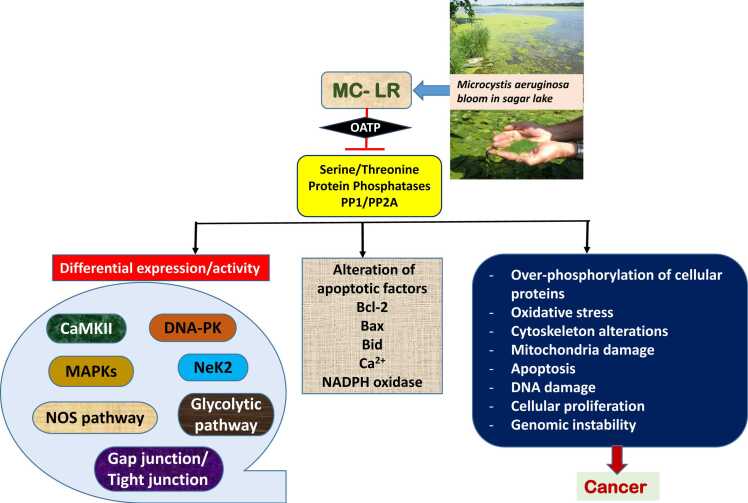
Illustrative outline of the potential mechanisms of microcystin-LR (MC-LR) toxicity: from absorption through organic anion transporting polypeptide channels (OATP) to serine/threonine protein phosphatase inhibition (PP1/PP2A) and cellular toxicity.

Cyanobacteria (CB) are photosynthetic cosmopolitan prokaryotic organisms that have been isolated from aquatic (freshwater, brackish and marine), terrestrial (soil, lichen-associated and the surface of leaves), and different aquatic and terrestrial extreme environments (hot springs, high salinity, deserts) [1]. These are responsible for the proliferation of toxic algal blooms that can harm aquatic, cattle, and human health [2]. Cyanobacterial bloom represents a significant hazard to human and ecological health in water bodies worldwide (Fig. 1). These blooms are responsible for the production of cyanotoxins, which have a detrimental effect on the drinking water in many areas [3]. Several cyanobacterial species produce cyanotoxins, which are released during cell death and can cause harm in 2 ways: extracellular secretion into the water or intracellular toxin via ingestion [4] (Table 1). CB multiplies at an exponential rate and creates a growing quantity of cyanotoxins as they are powered by an abundance of nutrients [5]. Cyanotoxins encompass various toxic compounds, including cyclic peptides and alkaloids. Among the cyclic peptides, microcystins and nodularins (NOD) are the most recognized, while the alkaloids category includes anatoxin-a, anatoxin-a(S), cylindrospermopsin (CYN), saxitoxins (STXs), aplysiatoxins, and lyngbyatoxins. Of these, most toxicity data are available for microcystin-LR (MC-LR), with only limited studies focusing on NOD and even fewer on alkaloids like anatoxin-a, CYN, and STX [6]. Microcystins (MCs), which are hepatotoxins, are produced by several cyanobacterial species and are increasingly prevalent due to climate change and human activities [7]. MC-LR targets hepatocytes via OATP1B1/1B3 transporters, inducing necrotic cell death, inflammation, and disrupted liver functions [8]. The first record of microcystin production was in 1942–43 from Microcystis toxic blooms in Vaal Dam reservoir, South Africa. Microcystin-LR (MC-LR) was first isolated and identified in the 1970s as a toxic compound produced by species of cyanobacteria, particularly from *Microcystis aeruginosa*. It was characterized for its significant hepatotoxic activity, leading to increased interest in cyanobacterial blooms and their effects on aquatic ecosystems and public health [9], [10]. The accumulation of cyanotoxins in aquatic organisms and their subsequent migration up the food chain to higher trophic levels endangers the health of animals and people from various genera which include *Microcystis, Dolichospermum, Oscillatoria*, *Planktothrix, Nostoc* and various other genera [11]. The increasing water hazards caused by these toxin-producing cyanobacteria are mostly found in eutrophic freshwater bodies, such as lakes, rivers, lagoons, streams and reservoirs. Among all these variants, as mentioned earlier, MC-LR is the most common and most studied congener, followed by MC-RR [12]. According to the World Health Organization (WHO) MC-LR levels in drinking water should not exceed 1 μg/L. Generally, exposure to MC-LR can affect fish and mammals on various levels, from biochemical changes to impacts on individual organisms. One major effect is oxidative stress, which disrupts redox signaling and regulation, potentially leading to molecular damage. Extensive research has shown that MC-LR exposure can cause liver damage, and even lead to hepatocarcinogenesis, as the liver is one of the primary organs affected. Furthermore, MC-LR inhibits protein phosphatase 1 and 2 A, increases reactive oxygen species (ROS) levels, and leads to cytoskeletal damage and hepatocyte necrosis [13] (Fig. 2).

**Table 1:** Comparison of MC-LR and other cyanotoxins, their primary organ target, health effects, toxin producing genera and mechanism of action.

**Toxin Type**	**Cyanotoxins**	**No. of known variants or analogues**	**Primary organ target(s)**	**Health effects**	**Toxin-producing genera**	**Mechanism of action**	**References**
Hepatotoxins	Microcystin-LR	80–90	Liver, Kidney, Brain, Heart	− Sore throat − Abdominal pain − Vomiting − Diarrhoea − Liver inflammation	*Microcystis, Dolichospermum, Nodularia,* *Nostoc, Oscillatoria, Planktothrix*	Inhibition of protein phosphatases 1, 2A and 3, tumor promoter, genotoxicity	[57]
	Cylindrospermopsin	3	Liver, Kidney, Lung, Spleen, Heart	− Acute pneumonia − Acute dermatitis − Kidney damage − Potential tumor growth	*Cylindrospermopsis, Dolichospermum, Rhaphidiopsis, Aphanizomenon*	Irreversible inhibition of protein and glutathione synthesis, Implicating cytochrome P−450	[28]
	Nodularin	6–8	Liver, Lung	− Abdominal pain − Myalgia	*Nodularia*	Inhibition of protein phosphatases 1,2A and 3, Tumor promoter	[52]
Neurotoxins	Anatoxin a	2–6	Nervous system, Lung	− Tingling, burning − Numbness − Salivation − Respiratory paralysis leading to death	*Dolichospermum, Planktothrix, Cylindrospermopsis, Oscillatoria*	Depolarizing neuromuscular blocking	[126]
	Saxitoxins	26	Nervous system	− Twitching − Respiratory arrest death	*Dolichospermum, Cylindrospermopsis, Aphanizomenon, Lyngbya*	Blocking neuronal communication by binding to the voltage-gated Na^2+^ channels	[127]
Dermatoxins	Aplysiatoxin	80	Skin	− Skin tumors − Itching	*Lyngbya, Oscillatoria, Schizothrix*	Potent tumor promoters and protein kinase C activators	[128]

In this review we attempt to provide a detailed overview of microcystins, focusing on MC-LR, one of the most toxic and widespread cyanotoxins found in our environment. By exploring MC-LR’s chemistry, transmission through air, water and food, and its toxic effects on various organs, this article brings together valuable knowledge that helps us understand how these toxins impact human and animal health. This review sheds light on how MC-LR enters biological systems, the mechanisms by which it damages cells, and its specific effects on vital organs. Highlighting these insights in one comprehensive source can guide future research and raise awareness about the risks associated with cyanotoxins, which is essential for developing better protective and treatment strategies. This research uniquely combines how MC-LR spreads, its toxic effects on organs, and its damage mechanisms, offering a full view of the health risks. It highlights critical insights to help us better understand and address cyanotoxin related health issues.

## 2 Chemistry of microcystin

The approximate molecular weight of microcystins is believed to be 1000 daltons. They are macrocyclic heptapeptides and the two terminal amino acids of the linear peptide are condensed (joined) to form a cyclic compound. A wide array of monocyclic peptide toxins, commonly referred to as MCs, comprises seven amino acids arranged in a ring structure along with a single amino acid side chain (ADDA group). These toxins have a total of one hundred known variants [14]. The nomenclature of each variant is computed from two variable positions where L-amino acids are consistently present. Some of the variants, like MC-LF (which has phenylalanine) and MC-LW (which has tryptophan), have hydrophobic amino acid residues, while MC-LR is more hydrophilic [15]. By replacing hydrophilic amino acids with hydrophobic ones, as observed in MC-LF and MC-LW, the hydrophobic nature of these variants is enhanced. The modifications in hydrophobicity have the potential to impact how MCs interact with proteins, cellular membranes, and other components of organisms, thereby potentially influencing their bioavailability and toxicity. Some unique amino acids found in microcystins, like Mdha (N-methyldehydroalanine) and D-MeAsp (D-erythro-β-methylaspartic acid), are essential for their bioactivity [16]. Mdha is a modified amino acid that comes from alanine. It features a double bond in its side chain, which gives it reactive properties. The unsaturated bond in Mdha enables it to create covalent bonds with specific target proteins, including protein phosphatases (PP1 and PP2A). This covalent interaction plays a crucial role in inhibiting these enzymes, which significantly contributes to the toxicity of microcystins. Mdha is instrumental in the toxin's capacity to interfere with cellular signaling and enhance hepatotoxicity. On the other hand, D-MeAsp is a modified form of aspartic acid that features an extra methyl group on the β-carbon. It is found in the D-configuration, which is less frequently seen in naturally occurring peptides [17]. The methyl group increases the structural rigidity and hydrophobic nature of the molecule, affecting how it interacts with target proteins. The stereochemistry (D-configuration) guarantees effective binding to the active sites of protein phosphatases, improving the selectivity and potency of microcystins. Additionally, the presence of D-MeAsp helps the toxin resist enzymatic degradation, extending its biological activity. MCs are chemically more stable due to their cyclic structure and novel amino acids, allowing them to stay in natural water for several months or years [18]. Due to this stability, more than 100 MC isoforms were identified, and their toxicity varied considerably. One of the most hazardous variants of microcystin is thought to be microcystin-LR (MC-LR). It interacts with the catalytic centers of serine/threonine protein phosphatases 1 and 2A (PP1 and PP2A), thereby resulting in the hyperphosphorylation of proteins, which disturbs the equilibrium and interferes with the many physiological activities of PP1 and PP2A [19].

**Fig. 3:**
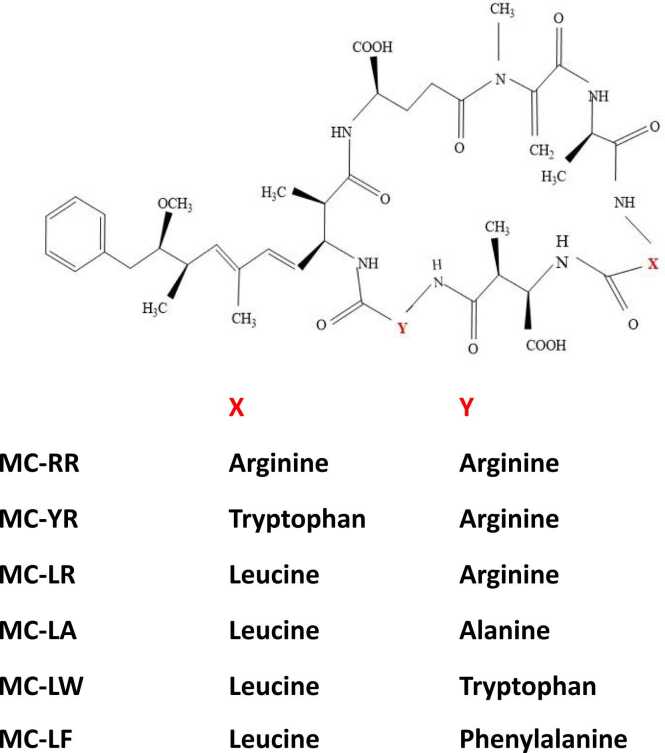
Chemical structure of microcystin (MC) and its variants.

MC-RR and MC-YR are two other variants of MCs that are quite common [20]. The toxicity is maintained when hydrophobic L-Leu at the first variable position is swapped out for another hydrophobic L-amino acid, such as alanine, phenylalanine, or tryptophan, but it is significantly reduced when a hydrophilic amino acid, such as arginine, is used in its place. Microcystins are denoted by the single-letter abbreviations for the amino acids at positions X and Y, respectively. Many of the MCs variants are caused by changes in the X and Y locations of amino acids. With diverse combinations of leucine (L), arginine (R), or tyrosine (Y), the most popular are MC-LR, MC-RR and MC-YR, with ADDA moiety present in all the forms [21] (Fig. 3).

## 3 Transmission of microcystin through various routes

MC can enter the bodies of humans through a variety of different entry points. The most direct way is by absorption of contaminated water, such as drinking contaminated water or engaging in recreational activities in polluted bodies of water. Consumption of microcystin-containing foods is a more indirect approach to absorbing MCs. When contaminated water is used to irrigate crops, MCs also make their way into the human food chain. There are few recorded occurrences of acute microcystin poisoning in humans. Nonetheless, a tragic instance in Brazil demonstrates the devastating impact of MCs on human health where patients undergoing hemodialysis were given improperly treated water [22].

### 3.1 Consumption of contaminated water (oral route or ingestion)

Drinking contaminated water exposes people to MCs most frequently, which has been extensively studied [23]. Depending on the toxin's composition and the stage of development of the cyanobacterial cell, cyanotoxins may be present intracellularly or as dissolved extracellular toxins in the surrounding water [24]. Low concentrations of MCs in water, which are consumed either accidentally or as part of daily drinking water requirements, harm human health [25]. Oral consumption of MCs at a dose concentration of 1–7 mg/kg bw results in severe toxicity after exposure [26]. The lysis of cyanobacterial cells, which occurs when polluted water intended for human consumption is boiled, might result in the release of more MCs [27]. After an algaecide was used to treat a cyanobacterial bloom in a nearby drinking water reservoir, a second epidemic broke out in Australia, resulting in the release of huge quantities of CYN [28]. The equilibrium between MC absorption, detoxification, and excretion determines the quantities and length of exposure, which in turn influence the severity of MC-induced toxicity. The most likely route of exposure is through the consumption of recreational water that contains CB accumulations [29]. Due to toxin biomagnification, direct ingestion by drinking water or devouring contaminated seafood remains the principal exposure route of concern [23]. Fish consumption is also a potential route of human exposure to the hepatotoxic MC-LR, especially in lakes and reservoirs that routinely experience significant toxic Microcystis blooms [30].

### 3.2 Body contact

Cutaneous exposure to aquatic cyanotoxins can occur during water-based recreational activities such as bathing and swimming. MCs are 200 times more poisonous than metal cyanide. The structural variations of this toxin include amino acid changes and modifications such as methylation and demethylation. Algal blooms can cause severe cases of dermatitis if they come into touch with the skin [31]. Interaction with CB in coastal marine environments has been linked to skin irritation and allergic reactions for at least 30 years. Humans may get exposed to cyanobacterial blooms during swimming, bathing or water-gliding in polluted water regions. The pollutants may come into touch with the body, causing rashes, itching and other allergic responses in the skin, eyes and ears [32]. The impact of these pollutants on the health of persons with atopic and nonatopic allergy illnesses remains uncertain, but it warrants the attention of researchers. Ten teen soldiers experienced mouth ulcers after coming into contact with *Microcystis aeruginosa* foam while participating in canoe-rolling and swimming drills in Rudyard Lake, Northwest England [33].

### 3.3 Inhalation

Inhaling CB-laced water spray may worsen preexisting respiratory diseases such as asthma [34]. The inhalation of sprays and mists that are produced by water sources that contain toxic CB is the mode of exposure that occurs in aquatic environments [35]. Humans may be exposed to MCs via inhalation which may be influenced by construction and military operations that destroy the soil structure of desert soils [36]. Coughing, a sore throat and hay fever are some of the specific respiratory reactions that have been linked to exposure to cyanobacterial bloom. This discovery implies that the inhalation of aerosolized CB compounds could potentially induce inflammatory reactions within the human body [37]. Furthermore, when MCs are absorbed through the respiratory system, they may affect a separate set of organs. Although MC may induce inflammatory responses in lung tissues, the toxin is rarely metabolized in the lungs when ingested or administered intraperitoneally [38]. Consumption of microcystins through inhalation at a dose concentration of 43 µg/kg bw on mice results in severe toxicity after the exposure [39]. Thus, inhaling an aerosol containing cyanobacterial cells or cyanotoxins is the only way to directly expose lung cells to microcystins.

### 3.4 Consumption of contaminated food

Numerous studies have revealed that MCs may be found in a wide variety of terrestrial and aquatic species that are consumed by humans even though these creatures come from different environments. By exposing French bean plants (*Phaseolus vulgaris*) to ambient concentrations of microcystin-LR in water, the entire leaf photosynthesis was inhibited, indicating infiltration of the toxin in the plants [40]. Consumption of MCs through contaminated food at a dose concentration of 5.95 µg results in severe toxicity after the exposure [41]. Cyanobacteria toxins collected or raised for human consumption must be considered in cyanobacterial toxin risk management. This might occur if agricultural plants are sprayed with water that contains cyanobacterial blooms and toxins [42]. A study investigating the microbial safety of commercial Spirulina products revealed that all the tested products contained MC at levels exceeding recommended daily limits [43]. Dairy cattle may consume water harboring cyanobacterial mass congregations and toxins, and in scenarios where dairy cow grassland may be inundated with toxins, it is important to consider the possibility that these toxins may be excreted into milk. Human exposure via food can result from consuming fish, plants, food supplements made of algae, or products with animal origins after using polluted water for agricultural or irrigation purposes [44].

According to scientific research, cyanotoxins can accumulate in dietary items to levels higher than the provisional limitations defined for MC-LR in drinking water to keep people from being exposed to it repeatedly. Many diverse aquatic species, such as fish, bivalve shellfish, and crustaceans may collect cyanotoxins by the absorption of CB cells or through the percutaneous route as dissolved poisons among the possibly contaminated food items [45]. Some edible crops have been shown to have cyanotoxins, which are thought to have been translocated to the plants via surface irrigation fluids. There is evidence that marine CB produces cyanotoxins, which are more often found in brackish water and estuaries, where aquaculture plants are often located because people eat more seafood than freshwater organisms. Liver and kidney diseases caused by MCs often have a financial effect on the meat and milk processing industries because these ailments can make animals less productive and smaller [46].

### 3.5 Uptake of algal dietary supplements

A significant portion of CB serves as a valuable reservoir of secondary natural products that find application in various sectors including agriculture, medicine, food, cosmetics, and energy [47]. The market for these goods, known as BGAS (blue-green algal supplements), have risen globally over the past few decades due to their purported health benefits. Due to the widespread propensity of planktonic CB to produce toxins, nutritional supplements containing cyanobacterial cells may serve as an exposure route in humans. The prolonged intake of CB dietary supplements has been linked to toxicity and liver damage, according to published publications [48]. One of the most effective ways to get exposed to MCs is through increased consumption of algal dietary supplements (ADS). ADS are naturally derived products that are distributed internationally and are widely accessible as capsules, tablets, or powders. They are promoted as being helpful to health and are easily accessible. ADS may assist improve mood, weight loss, energy, alertness, and detoxification, and it can also be employed in pharmaceutical therapy [49]. The Oregon Health Division and the Oregon Department of Agriculture have set a regulatory threshold of 1 µg/g for MCs in goods containing blue-green algae (BGA) and a concentration > 1 µg/g of MCs results in severe toxicity associated with BGAS [50].

## 4 Mechanism of toxicity

### 4.1 Interaction with protein phosphatases PP1 and PP2A

MCs have been scientifically shown to be very toxic to both animals and humans, causing immediate and long-term harm. Different microcystin variants' fatal dose (LD50) ranges from 50 to 1000 µg/kg bw [51]. MC-LR, inhibit the activity of protein phosphatases PP1 and PP2A, which are critical enzymes involved in cell regulation (Fig. 2). The inhibition of PP2A is considered a primary toxicity mechanism for MCs, but MCs impact PP2A not only through direct inhibition but also by regulating its expression through epigenetic and post-translational modifications such as methylation and demethylation [52]. The activity and substrate specificity of PP2A are influenced by the methylation status of its catalytic subunit (C-subunit). This methylation takes place at the C-terminal leucine residue of the PP2A catalytic subunit and is facilitated by leucine carboxyl methyltransferase-1 (LCMT-1). The demethylation of PP2A is facilitated by the action of protein phosphatase methylesterase-1 (PME-1). MCs can disrupt the normal processes of methylation and demethylation in PP2A. By attaching to the catalytic subunit, they may change its structure and accessibility, which in turn interferes with the functions of LCMT-1 and PME-1 [53]. This interference impacts the assembly and stability of PP2A holoenzymes, as methylation plays a vital role in attracting specific regulatory subunits. Such disruption further undermines the enzyme's functional diversity and regulatory functions. This inhibition leads to complex cellular effects, often involving a biphasic response in terms of MC concentration and exposure time. [54]. MC has a substantial affinity for protein phosphatases PP1 and PP2A, despite having a very insignificant effect on PP2B [55]. Because MC-LR binds to both PP1 and PP2A, it is possible to have a comprehensive understanding of both the structures and their enzyme inhibition. The cyclic peptide known as MC-LR synchronizes with the two metal atoms that are found in the phosphatase by attaching the water molecules to its carboxyl group, which helps it to coordinate with the water molecules. In addition to the active site of the enzyme, the MC-LR is capable of communicating with the surface pocket of PP2A, which is located above the two manganese atoms [56]. The interactions between the ADDA and the residue of the binding pocket increase the MC-LR binding. Several van der Waals forces facilitate the interaction between the hydrophobic region of MC-LR and the opposite end of the PP2A binding pocket. An increase in PP1/PP2A activity has been linked to acute toxicity induced by the MC on the liver tissue and the cytoskeleton elements [57]. Acute exposure to cyanotoxins is rare and usually does not lead to death. However, it is not prudent to disregard the long-term effects of cyanotoxins on the function of mammalian organs. Elevated mortality and chronic exposure to trace amounts of MCs in potable water have been linked to liver cancer [58].

### 4.2 Oxidative stress

The existence of reactive oxygen species (ROS), in addition to the poisonous chemical, is another factor that can subsidize the toxicity of MC. It is well established that mitochondria are the most susceptible target for MCs which is mostly associated with mitochondrial metabolism and can induce necrosis or apoptosis in addition to genotoxicity [59]. The principal functions of mitochondria are the electron transport chain (ETC) and oxidative phosphorylation (OXPHOS), a process involving five multiprotein complexes. The ETC in the mitochondria is thought to be a major source of ROS inside the cell, especially at the complex I and III levels [60].

**Fig. 4:**
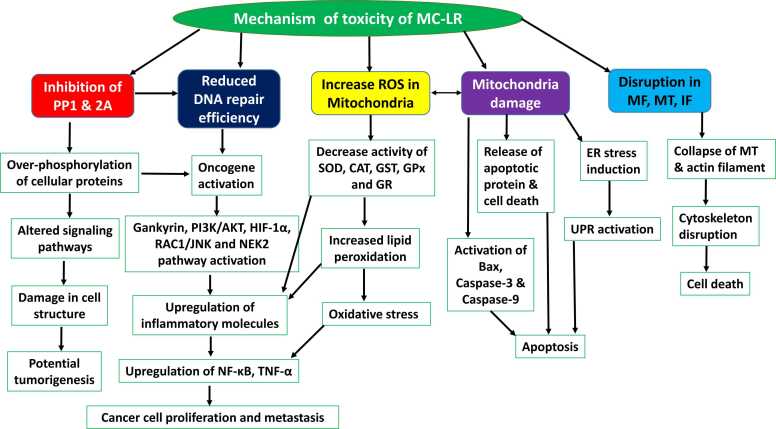
The figure shows the mechanisms of toxicity of MC-LR. Key pathways of mechanisms of toxicity of MC-LR include interaction with protein phosphatases (PP1 and PP2A), oxidative stress, MC-LR-induced oncogenic pathway, apoptosis pathways, and cytoskeletal disruption.

A recent study showed that MC-LR exposure increases oxidative stress by disrupting antioxidant defenses, evidenced by decreased activities of CAT, GST, SOD1, GPx, GR and increased lipid peroxidation (MDA) (Fig. 4). When autophagy is inhibited, oxidative damage and inflammation are intensified, raising levels of inflammatory markers like TNFα and IL11, and further weakening cell viability. Although autophagy inhibition reduces apoptosis markers, it leads to greater oxidative and inflammatory stress, ultimately harming cells more severely [61]. One more similar study revealed that MC-LR exposure in lung cells triggers oxidative stress by generating excessive reactive oxygen species (ROS) and reducing antioxidant defenses (SOD and GSH-Px). This imbalance leads to inflammation and cell death, as shown by increased levels of inflammation-related proteins (p-65, iNOS) and apoptosis markers (bax, cyt-c, caspase-9). Together, these factors contribute to lung damage and pulmonary toxicity [62]. MC-LR exposure in rice cells at environmentally relevant concentrations (0.05–50 μg/L) induces oxidative stress, damaging cell viability and increasing lipid peroxidation, as shown by high malondialdehyde (MDA) levels. Oxidative stress also elevates reactive oxygen species (ROS), including superoxide radicals. The rice cells respond with antioxidant defenses, activating enzymes like peroxidase (POD) initially and then superoxide dismutase (T-SOD), glutathione (GSH), and Glutathione-S transferase (GST) over time, highlighting a time- and dose-dependent adaptation to MC-LR's oxidative damage [63]. One more study support that MC-LR exposure increases cell damage, reactive oxygen species (ROS), and inflammatory markers like TNF-α and IL-6, while reducing antioxidant defenses like superoxide dismutase (SOD) and glutathione (GSH) [64]. In kidney cells MC-LR exposure leads to oxidative stress, evidenced by increased reactive oxygen species (ROS), higher malondialdehyde (MDA) levels, reduced glutathione (GSH), and changes in antioxidant enzymes. These effects were more significant at higher doses [65]. In HepG2 cells MC-LR induces oxidative stress by disrupting biochemical processes. It lowers antioxidant enzymes (superoxide dismutase and catalase), reduces glutathione, and increases malondialdehyde levels. This leads to cell damage and activation of oxidative stress pathways. The study also highlights the involvement of p53/p21WAF1/CIP1 signaling and the proto-oncogene c-myc in MC-LR-induced apoptosis, contributing to a better understanding of MC-LR's hepatotoxic effects [66].

### 4.3 MC-LR induced oncogenic pathway

MC-LR disrupts DNA repair pathways, leading to genotoxic effects. It inhibits the DNA repair enzymes PP1 and PP2A, reducing repair efficiency and increasing DNA damage, particularly in the liver. Additionally, MC-LR activates the protein CaMKII, which escalates oxidative stress and cell death processes, while also influencing proteins like Nek2 and P53, which are linked to cell growth and tumor suppression. This toxin further promotes the degradation of anti-apoptotic proteins like Bcl-2 and activates MAPKs, leading to uncontrolled cell proliferation and potentially promoting tumor development [67]. A recent study is showing that MC-LR is associated with cancer progression through multiple pathways (Fig. 2). It induces oxidative stress and DNA damage by increasing reactive oxygen species (ROS), causing mutations and promoting hepatocellular carcinogenesis. MC-LR also triggers epigenetic changes, notably DNA methylation, which silences tumor suppressor genes, supporting malignancy. It inhibits protein phosphatase 2A (PP2A), a tumor suppressor, leading to uncontrolled cell growth [68]. In colorectal cancer (CRC), MC-LR influences the tumor microenvironment by upregulating TGF-β1 in M2 macrophages while suppressing CST3, enhancing CRC cell migration and invasion [69]. Additionally, MC-LR stimulates tumor-associated macrophages to secrete CXCL1, which interacts with IGHG1, boosting CRC cell survival and migration [70]. The toxin also promotes CRC cell proliferation by upregulating HOXB4 and cadherin-11, fostering cell migration and invasiveness [71], [72]. MC-LR activates inflammatory pathways, increasing pro-inflammatory molecules like NF-κB, COX-2, and TNF-α, which support chronic inflammation and hepatocarcinogenesis [73]. Finally, MC-LR activates the IRE1α/XBP1/HK2 signaling pathway, enhancing glycolysis and lactate production, which further polarizes macrophages to a tumor-supportive M2 phenotype [74]. Collectively, these mechanisms illustrate how MC-LR facilitates tumor initiation, progression, and metastasis (Fig. 2, Fig. 4).

### 4.4 MC-LR induced apoptosis pathways

MC-LR can induce apoptosis, or programmed cell death, through both mitochondrial and endoplasmic reticulum (ER) pathways. Mitochondrial damage leads to the release of apoptotic proteins into the cytosol, activating caspases that break down cytoskeletal components. In the ER pathway, stress triggers the unfolded protein response, further leading to cellular breakdown and cytoskeletal disintegration [75]. A study showed that MC-LR exposure triggers an increase in ROS, leading to oxidative stress in cells, which can damage DNA, proteins, and other cell components. The oxidative stress caused by ROS stimulates specific genes (p38, JNKa and bcl-2) that play roles in programmed cell death (apoptosis). After exposure to MC-LR, the number of cells undergoing apoptosis (dying in a controlled way) in the liver increases significantly within 12–48 hours of exposure [76]. In another similar study the researchers found that MC-LR exposure specifically induced cell death in the liver but not in other organs like the kidneys or heart. MC-LR triggered apoptosis in liver cells. This cell death appeared to be linked to a significant reduction in the protein α-tubulin (which is essential for maintaining cell structure).

Additionally, two cellular pathways, p38-MAPK and CaMKII, were highly activated in the liver, which may contribute to apoptosis and tissue damage [77]. Another study is proving that higher MC-LR doses significantly triggered apoptosis in kidney cells, aligning with increased toxicity [78]. This study investigated MC-LR’s impact on the maturation of porcine (pig) oocytes and the mechanisms behind its toxicity. Oocytes exposed to 60 μM MC-LR showed decreased mitochondrial membrane potential (MMP) and an increased rate of early apoptosis, indicating cellular distress. This apoptotic response was further supported by reduced expression of protein phosphatase PP2A and increased expression of pro-apoptotic markers like p53, BAX, caspase3, and cleaved-caspase3. Collectively, these findings reveal that MC-LR induces oxidative stress, mitochondrial dysfunction, spindle defects, and apoptosis in porcine oocytes, ultimately impairing their maturation. This suggests a potential risk of reproductive toxicity due to MC-LR in mammalian oocytes [79]. In a similar study, researchers examined how MC-LR exposure affects lipid metabolism and apoptosis (programmed cell death) in the livers of female zebrafish (*Danio rerio*). The results showed that MC-LR alone led to liver damage marked by fat accumulation and increased apoptosis, shown by higher levels of triglycerides and the activation of genes associated with fat production (foxo1a, elovl5, pparγ) and apoptosis (bax, casp3) [80]. In human umbilical vein endothelial cells (HUVECs), MC-LR increased cell death by activating key apoptotic pathways, including mitochondrial damage, reactive oxygen species production, and changes in key proteins like p53 and PCNA. This suggests that MC-LR promotes cell death through mitochondrial signaling, leading to vascular dysfunction and malformations [81] (Fig. 4).

### 4.5 MC-LR induced cytoskeletal disruption

The cytoskeleton is like the cell's scaffolding, providing structure and shape, and helping with transport within the cell and a study revealed that MC-LR’s ROS production harms this structure, particularly affecting microtubules (MTs) (key parts of the cytoskeleton) [76]. MC-LR disrupts all major cytoskeletal components-microfilaments (MFs), MTs, and intermediate filaments. It causes MTs and actin filaments to collapse toward the cell nucleus, while intermediate filaments such as keratin and vimentin undergo hyper phosphorylation, leading to their disorganization. This rearrangement impairs cellular structure, resulting in membrane blebbing, cell rounding and eventually cell death [75] (Fig. 4).

One more similar study revealed that MC-LR exposure disrupted the liver cells’ cytoskeleton by reducing α-tubulin levels by almost half (45.56 % of the levels seen in untreated mice). The cytoskeleton is critical for cell shape, structure and function, so its disruption likely contributes to cellular damage and death [77]. One more study showed that MC-LR caused MFs and MTs in kidney cells to collapse, leading to partial or complete cytoskeletal breakdown. Changes were seen at the gene level as well, with shifts in the expression of genes like β-actin, lc3a, and keratin, which are linked to cytoskeletal structure and stability [78]. In a study, hepatocytes and other cell types were exposed to MC-LR to investigate how it affects each cytoskeletal component over time and at varying concentrations. Results showed that MC-LR disrupts the cytoskeleton in liver cells (hepatocytes) by inhibiting serine/threonine protein phosphatases, essential enzymes that regulate cytoskeletal stability [82]. This disruption primarily affects three main cytoskeletal structures: actin microfilaments, intermediate filaments (IFs) and MT. MC-LR first cause’s disorganization of IFs, followed quickly by MT disruption, leading to the collapse of both structures around the cell nucleus. In hepatocytes, MT changes often occur before IF disruption, while MFs are altered later in the sequence, initially clustering under the cell membrane, forming rosette-like shapes, and eventually condensing around the nucleus. These findings suggest a shared mechanism across cell types, although distinct changes in hepatocytes indicate there may be multiple phosphorylation sites involved in disrupting the cytoskeletal components [83] (Fig. 4).

## 5 Toxicological effect of microcystin on different organs

**Fig. 5:**
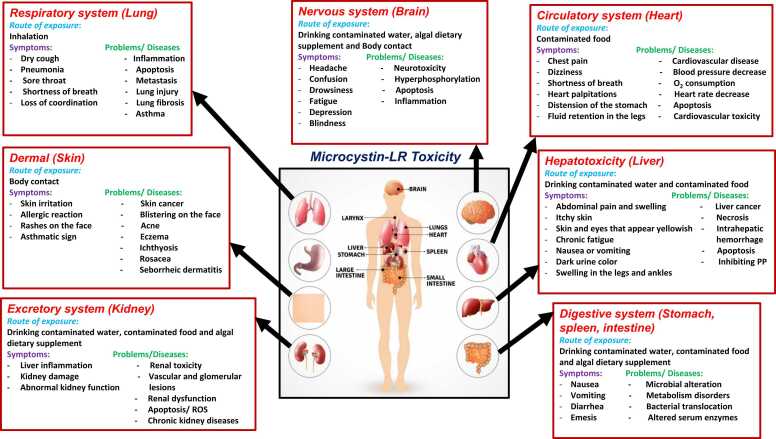
Effect of microcystin toxicity on various organs of mammals.

Microcystin exposure can alter the functionality of living organisms by disturbing their overall health. MCs can infiltrate the colon and bloodstream and then spread to a variety of organs, such as the liver, brain, kidney, lung, heart, and sexual organs, after being exposed and assimilated into the blood circulation [84] (Fig. 5). Apoptosis is the unidirectional response of cells and tissues to toxins which is accompanied by cell shrinkage, chromatin condensation, plasma membrane blebbing, oligonucleosomal DNA breaking and cell disintegration (apoptotic bodies) [85].

### 5.1 Liver

The liver is the primary organ that is targeted by MCs. During intoxication, the organic anion transporting peptides (OATP1B1, OATP1B3, and OATP1A2) and bile acid transportation system are accountable for the absorption of MCs by hepatocytes [86]. OATP1B1 and OATP1B3 are members of the liver-specific subfamily of OATPs, which are confined to the sinusoidal membrane of hepatocytes and transport a vast array of therapeutically relevant medicines [87]. The tissue distribution of the MC-LR transporting OATPs that have been identified provides a comprehensive explanation for the targeted organ toxicity of MCs in the liver and brain. MC-LR uptake into rat and human hepatocytes is mediated by OATP1B1 and OATP1B3, respectively, whereas OATP1A2 facilitates MC-LR transport across the blood-brain barrier [88]. When the liver fails to adequately detoxify and metabolize both endogenous and exogenous toxins, it may be accompanied by an aggregation of blood in the liver, resulting in an enlargement of the liver. Since MCs have a strong affinity for the liver, their hepatotoxicity has received a great deal of attention [89], [90], [91], [92], [93]. Oxidative damage and the production of reactive oxygen species (ROS) are impacted by disturbances in the maintenance of cellular iron homeostasis. MC exposure at a concentration of 20 µg/L is responsible for hepatotoxicity in various animal models [92]. In contrast, higher dosages of MC-LR cause perforation of the mitochondrial membrane, extensive intrahepatic bleeding, as well as hepatic architectural injury, all of which are indicative of cytoskeletal disruption in the liver [94]. Hepatotoxicity is caused by MCs and NOD penetrating hepatocytes, where they stimulate apoptosis of liver cells and hyperphosphorylation of proteins within the liver [95]. Hepatocyte degeneration, cytotoxicity, necrosis, non-alcoholic steatosis, intrahepatic hemorrhage, hepatocellular carcinoma, and even animal death may result from microcystin's ability to irreversibly suppress the activities of PP1 and PP2A in hepatocytes [96] (Fig. 5).

### 5.2 Brain

Neurotoxicity is the term used to describe the detrimental impact that poisonous substances have on the regular operation of the nervous system. It is a prominent factor in the development of neurological disorders. The accumulation of MCs in the brain can result in hyperphosphorylation, neuronal injury, inflammation, and cell death [97]. MC-LR and MC-RR have been discovered in the brain of fish, and they may elicit neurotoxic effects via traversing the blood-brain barrier [98]. Microcystin exposure at a concentration of 20 µg/L is responsible for the neurotoxicity in various animal models [99]. The neurotoxic consequences of MCs encompass neurostructural, physiological and behavioral aspects, including deficiencies in power of retention, nervousness, mental trauma, disorientation, self-hood, headaches, vertigo and nausea, as well as vision impairment and blindness [100]. Prolonged exposure to MC-LR at levels below fatal doses resulted in reduced swimming activity in two fish species (*Danio rerio* and *Leucaspius delineatus*). This reduction in activity was measured using an automated video-monitoring and object-tracing system, which detected neuro-functional changes [101]. This indicates that there is a significant possibility that MC-LR could result in adverse effects on fish populations, including reproduction and predator-prey interactions. Cholinergic system regulation of neurotransmitter activity is among the most critical, as it is responsible for cognitive and locomotor function control [102]. MCs may influence acetylcholinesterase (AChE), which has historical roles in stopping neurotransmission at cholinergic synapses and neuromuscular junctions and which was elevated in zebrafish brain after MC-LR exposure [45] (Fig. 5).

### 5.3 Kidney

The cortex and medulla of the kidney might suffer harm when it is subjected to MCs for extended periods at relatively modest dosages [103]. MCs increase renal changes and influence renal physiology and renal damage in rats exposed to MCs [52]. Oxidative stress and cytoskeletal instability may combine and simultaneously result in MC-induced apoptosis and kidney damage [104]. Damage to the kidneys brought on by MCs will result in the loss of the organ's ability to filter waste materials out of the blood, regulate blood pH, and produce hormones. Additionally, the kidney's extraordinary capacity to heal damaged nephrons and direct renal regeneration might also be impacted [105]. MC exposure at a concentration of 10 µg/kg bw is responsible for the nephrotoxicity in various animal models [106]. MC-LR has the potential to accumulate in the kidney and has been demonstrated to induce nephrotoxicity. Also, long-term chronic kidney disease causes an iron metabolism disorder that makes it hard for red blood cells to differentiate. This, along with a drop in renal erythropoietin, leads to severe anemia in CKD patients [107]. The term "chronic kidney disease of undetermined etiology" (CKDu) refers to a novel type of chronic kidney disease that has no recognized cause, such as diabetes, chronic glomerulonephritis, or hypertension [108] (Fig. 5).

### 5.4 Lung

The liver damage caused by MCs can create and release inflammatory mediators that induce subsequent lung damage [109]. Microcystin exposure at a concentration of 40 µg/L is responsible for lung toxicity in various animal models [110]. It has also been found that MCs can cause apoptosis in lung tissue and inflammation, as well as a significant rise in lung impedance [83]. Tight junctions, which are necessary for lung gas exchange, cancer metastasis and embryonic development, can be influenced by MC-LR [111]. The pulmonary system must provide a precise barrier between the external environment and the fluid-filled tissues to facilitate gas exchange. An elevation in the concentration of MC-LR within the respiratory tract is accompanied by hypoxic regions and a thickening of the alveolar septa. Nevertheless, mounting evidence suggests that the lung may be another crucial target organ for MCs, which can have both acute and chronic effects on the lung when injected intravenously or intraperitoneally [112]. MC-LR might alter the anatomy of the lungs and may influence respiratory function. Alveolar epithelial cells can form a variety of intercellular connections. The epithelial barrier function depends mostly on the tight connection among them [113]. In addition, it has been postulated that a liver that has been damaged will produce and release inflammatory cells, which will then cause secondary lung injury. This happens specifically when blood is delivered to the pulmonary artery from the posterior vena cava, which is nourished by the hepatocyte portal vein. Reactive oxygen species (ROS), which are created by defense cells such as neutrophils, monocytes, and macrophages when they are activated due to MC-LR, can cause damage to the lung tissue during an inflammatory phase [110] (Fig. 5).

### 5.5 Small intestine

MCs also aim to infect other organs in the body, including the digestive system. MCs accumulating in the gut can have harmful consequences on the gastrointestinal system [114]. MC-LR is frequently found deposited in the mucous layer of microvilli and mucus that is released by the mucosal epithelial cells of the small intestine, as well as in the cytoplasm and in the region around the nucleus [115]. Microcystin exposure at a concentration of 50 µM is responsible for intestinal toxicity in various animal models [115]. MCs produce gastrointestinal toxicity by changing the function of digestive enzymes in the intestinal chorion, inducing oxidative stress and cell death in enterocytes, or altering the intestinal microbiota [116]. Oral consumption of MC-LR has the potential to cause histological damage to the gastrointestinal tract where intestinal villi capillaries can allow it to enter the bloodstream [117]. The small intestine is made up of the duodenum, the jejunum, and the ileum. MCs accumulate rapidly in the colon but are eliminated slowly, possibly because covalently bound MCs are present in the intestine [116]. Additionally, MC-LR can cause the ileum, jejunum, and duodenum to undergo apoptosis, which can cause gastroenteritis, resulting in diarrhea and discomfort in the abdomen [114] (Fig. 5).

### 5.6 Heart

When MCs travel through the circulation and are delivered to the heart, there is a potential for cardiac damage to occur. Microcystin exposure at a concentration of 10 µg/kg bw is responsible for cardiotoxicity in various animal models [118]. Pathophysiological changes caused by MCs include a decline in pulse rate and cardiac function, hypotension in response to whole blood volume expansion, a decrease in pulse rate and arterial pressure, a reduction in oxygen utilization and the mobilization of splenic and hepatic blood reserves [117]. Being the first organ to develop during fetal development, the heart nourishes and oxygenates all other organs and tissues. Normal cardiovascular system functions are primarily impacted by hereditary variables, environmental factors, and their interaction, which is recognized as a significant cause of cardiovascular disease (CVD) [119]. This suggests that MCs may directly impact the heart's tissues and the circulatory system, leading to abnormalities in the cardiovascular system's structure and/or operation. Cardiovascular tissue is made up of many different types of cells, including cardiac cells, fibroblasts, mast cells, endothelial cells, white blood cells, as well as other immunological cells [120]. MCs may potentially promote cardiovascular damage by altering the shape of cardiomyocytes and the respiratory chain enzyme activity in cardiomyocyte mitochondria. The heart is known to include a large number of blood vessels, as these vessels are responsible for blood flow. MC-LR may cause cardiovascular toxicity by changing the shape of cardiomyocytes, the rate of cell growth and death, the cytoskeleton, and the rhythm of the cells. Furthermore, these substances possess the ability to modify the membrane potential, ultrastructure, oxidative stress levels, and enzyme activity of the respiratory chain within cardiomyocyte mitochondria [121] (Fig. 5).

### 5.7 Reproductive system

A low dose of MC-LR may cause severe harm to the male reproductive system which includes testicular injury and a decline in sperm quality. Comparing mammals to aquatic species, MCs are less likely to build up in the gonads of mammals. Microcystin exposure at a concentration of 10 µg/kg bw is responsible for reproductive toxicity in various animal models [60]. MCs can cause damage to the testis, a reduction in sperm quality and viability, and abnormalities in sperm, and blood flow to the testis. The buildup of MCs in the gonads (ovary) of zebrafish (*Danio rerio*) has been testified to produce direct reproductive damage, which can retard ovarian maturation, reduce ovary weight, reduce the number of primordial follicles, disrupt the estrus cycle, and lower progesterone levels [122]. Necrosis, hyperplasia, inflammation, and fibrosis of the prostate are all signals of MCs which upon accumulation can impede the growth of the prostate in the progeny [123]. The prostate is a significant sex accessory gland in male mammals. It can release prostatic fluid, which is one of the essential components of sperm and hence plays a crucial part in male reproduction. The improper growth of the prostate will substantially impair the operation of the male reproductive system [123]. Damage to the gonads (testes, ovaries, and prostate) from MC-LR can lead to poor sperm quality and disruptions in the balance of sex hormones in the body.

### 5.8 Colon

MCs may contribute to the growth of cancer in the colon and rectum [124]. Colorectal cancer, which is quickly increasing in nations like China, the United States, and Australia, is another malignancy associated with MCs [115]. Unsuspected risk factors for colorectal cancer encompass dietary patterns, insufficient physical activity, and overweight status. Colorectal cancer ranks as the fourth most prevalent malignancy among men and the third most prevalent malignancy among women on a global scale [125]. Microcystin-LR exposure at a concentration of 50 µM is responsible for colon-related toxicity in various animal models and cell lines [115].

## 6 Conclusion

Due to rising water eutrophication, microcystins are now generally recognized as an environmental hazard that poses a significant threat to human health, especially with chronic exposure. This review comprises the existing knowledge on MC-LR, highlighting its chemistry, transmission pathways, mechanisms of toxicity and impacts on various organ systems. By different exposure routes such as ingestion of contaminated water, dermal contact, inhalation, and consumption of contaminated food or algal supplements, humans can be exposed to MC-LR. MC-LR primarily causes toxicity through inhibition of protein phosphatases PP1 and PP2A which leads to disruptions in cellular signalling pathways and oxidative stress**.** These toxic effects further induce oncogenesis, apoptosis, and cytoskeletal disruption, ultimately compromising cellular integrity and function**.** MC-LR has toxicological impacts on major organ systems, particularly the liver, where it accumulates and exerts potent hepatotoxic effects. MC-LR exposure is also associated with neurotoxicity, nephrotoxicity, and adverse impacts on the lungs, intestines, heart, reproductive system and colon, with varying degrees of damage depending on the level and duration of exposure.

## CRediT authorship contribution statement

**Rajpoot Roshni:** Writing – original draft, Investigation, Formal analysis, Data curation, Conceptualization. **Rajput Siddharth:** Writing – original draft, Investigation, Formal analysis, Data curation. **Koiri Raj Kumar:** Writing – review & editing, Supervision, Resources, Project administration, Investigation, Funding acquisition, Conceptualization.

## Declaration of Competing Interest

The authors declare that they have no known competing financial interests or personal relationships that could have appeared to influence the work reported in this paper.

## Data Availability

No data was used for the research described in the article.
